# Age related changes in striatal resting state functional connectivity in autism

**DOI:** 10.3389/fnhum.2013.00814

**Published:** 2013-11-28

**Authors:** Aarthi Padmanabhan, Andrew Lynn, William Foran, Beatriz Luna, Kirsten O'Hearn

**Affiliations:** Laboratory of Neurocognitive Development, Department of Psychiatry, University of PittsburghPittsburgh, PA, USA

**Keywords:** autism, fMRI, resting state, functional connectivity, striatum, development

## Abstract

Characterizing the nature of developmental change is critical to understanding the mechanisms that are impaired in complex neurodevelopment disorders such as autism spectrum disorder (ASD) and, pragmatically, may allow us to pinpoint periods of plasticity when interventions are particularly useful. Although aberrant brain development has long been theorized as a characteristic feature of ASD, the neural substrates have been difficult to characterize, in part due to a lack of developmental data and to performance confounds. To address these issues, we examined the development of intrinsic functional connectivity, with resting state fMRI from late childhood to early adulthood (8–36 years), using a seed based functional connectivity method with the striatal regions. Overall, we found that both groups show decreases in cortico-striatal circuits over age. However, when controlling for age, ASD participants showed increased connectivity with parietal cortex and decreased connectivity with prefrontal cortex relative to typically developed (TD) participants. In addition, ASD participants showed aberrant age-related connectivity with anterior aspects of cerebellum, and posterior temporal regions (e.g., fusiform gyrus, inferior and superior temporal gyri). In sum, we found prominent differences in the development of striatal connectivity in ASD, most notably, atypical development of connectivity in striatal networks that may underlie cognitive and social reward processing. Our findings highlight the need to identify the biological mechanisms of perturbations in brain reorganization over development, which may also help clarify discrepant findings in the literature.

## Introduction

A recent focus in autism research is the differences in functional connectivity in autism spectrum disorder (ASD). Early studies suggested that functional connectivity was altered in ASD during tasks examining executive function (Koshino et al., 2005, 2008; Just et al., 2007; Mostofsky et al., 2009), language (Just et al., 2004; Kana et al., 2006), face processing (Kleinhans et al., 2008), social-emotion processing (Welchew et al., 2005; Rudie et al., 2012b), selective attention (Keehn et al., 2013), and visuomotor coordination (Mizuno et al., 2006; Mostofsky et al., 2009). These findings have led to the strong hypothesis that ASD is a “disorder of abnormal brain connectivity” (Belmonte et al., 2004; Frith, 2004; Just et al., 2004; Geschwind and Levitt, 2007; Hughes, 2007; Minshew and Williams, 2007; Ecker et al., 2013), with the predominant theory being that hypo-connectivity, was core to the pathophysiology of the disorder. However, results from task-related functional connectivity studies have been mixed as reports indicate both hypo- and hyper- functional connectivity in ASD relative to typically developing (TD) individuals (Muller et al., 2011).

**Figure 1:**
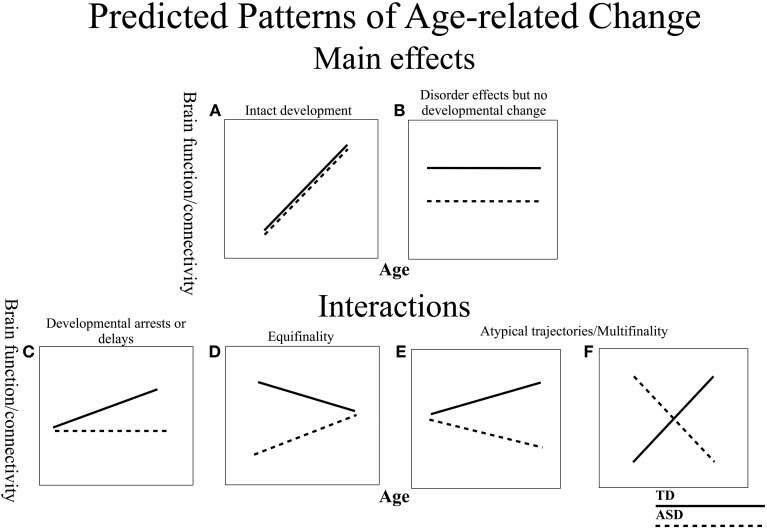
**Schematic of predicted models of age-related change in ASD and TD**. The ASD group is depicted with dashed lines and the TD with solid lines. **(A)** Both groups display age-related change, but do not differ in trajectories suggesting intact development in ASD. **(B)** Stable disorder effects that persist over development, with no age-related changes. **(C)** TD group shows age-related change, but ASD group remains unchanged, suggesting a developmental arrest or delay. **(D)** Both groups display differential developmental trajectories that converge into adulthood (Equifinality). **(E,F)** Both groups display differential developmental trajectories that diverge in adulthood (Multifinality).

Examining resting state functional connectivity may help address some of the discrepant findings, as it provides a measure of intrinsic functional connectivity without the task-related differences that confound comparisons across different age and clinical groups. However, similar to the task-based literature, much of the prior resting state connectivity research testing individuals with ASD has suggested overall decreases in intrinsic connectivity (Cherkassky et al., 2006; Kennedy and Courchesne, 2008; Monk et al., 2009; Assaf et al., 2010; Jones et al., 2010; Weng et al., 2010; Anderson et al., 2011; Dinstein et al., 2011; Ebisch et al., 2011; Gotts et al., 2012; Rudie et al., 2012a; Mueller et al., 2013; Tyszka et al., 2013; von dem Hagen et al., 2013), while some others have found increases (Noonan et al., 2009; Di Martino et al., 2011; Delmonte et al., 2013; Lynch et al., 2013; Uddin et al., 2013a; Washington et al., 2013). Taken together, these findings suggest that the nature of connectivity differences in ASD is not yet fully characterized. Inconsistent findings in functional connectivity might be due to many important differences between various studies (e.g., data acquisition and analysis, small sample sizes, diagnostic criteria). It is especially likely that differences in age ranges and a lack of examination of developmental changes contribute to conflicting reports in the literature (for review, see Uddin et al., 2013b). In general, developmental work in ASD is limited, with a few prior studies examining age-related change in gray matter (Langen et al., 2009; Greimel et al., 2013), structural connectivity (Kleinhans et al., 2012), behavior (Luna et al., 2007), task modulated brain function (Schulte-Ruther et al., 2013), and resting state connectivity with the default mode network (Wiggins et al., 2011). The majority of these studies report atypical trajectories in ASD. Given that (1) typical connectivity develops substantially, with connectivity patterns changing well into adulthood (Fair et al., 2009; Hwang and Luna, 2011), (2) group differences may manifest differently at different time points over the lifespan, and (3) ASD is characterized as a disorder of abnormal and delayed development in the brain, there is a strong possibility that functional connectivity matures differently in ASD. Thus, examining age-related changes in brain connectivity is crucial to understanding the neural basis of ASD. Importantly, there is a need to clarify the nature of developmental change (Figure 1). For example, developmental abnormalities can be classified as arrested (a lack of development) (Figure 1C), or atypical (a deviating developmental trajectory) (Figures 1D–F). In addition, it is important to know if regions show intact development in ASD (Figure 1A), or no developmental changes, but show persistent disorder effects (Figure 1B). Characterizing these patterns is essential to understanding the progression of the disorder and for identifying time points of plasticity and/or vulnerabilities.

One particularly understudied topic in the ASD literature is differences in connectivity with the striatum, an important subcortical region associated with a number of core cognitive and affective functions (Postuma and Dagher, 2006) that are atypical in ASD-including social reward processing. The striatum, which includes the caudate, putamen, and nucleus accumbens bilaterally, has extensive connections to cortex and cerebellum via the thalamus. Prior studies of functional connectivity during rest in typical adults have revealed a number of functionally distinct but overlapping cortico-striatal circuits (Di Martino et al., 2008; Kelly et al., 2009b; Furman et al., 2011) that underlie core motor, cognitive, affective, and reward processes. Important for the current study, neuroimaging research has found structural and functional differences in ASD in the striatum. Structural neuroimaging studies have shown differences in striatal volume in both children and adults with ASD, specifically in the caudate nucleus (Stanfield et al., 2008; Langen et al., 2009; Qiu et al., 2010; Estes et al., 2011). Functional neuroimaging studies demonstrate striatal activation differences in children, adolescents, and adults with ASD (in separate studies) during tasks of sensorimotor control and higher order cognition (Schmitz et al., 2006; Takarae et al., 2007; Shafritz et al., 2008), social processing (Dapretto et al., 2006; Masten et al., 2011; Weng et al., 2011; Delmonte et al., 2012), and reward processing (Scott-Van Zeeland et al., 2010; Delmonte et al., 2012). Diffusion tensor imaging studies have previously shown decreased white matter connectivity between striatum and prefrontal cortex ASD relative to TD (Langen et al., 2011) or no group differences in adults (Delmonte et al., 2013). With regards to functional connectivity with striatum, most prior research has suggested hyper-connectivity. For example, Turner et al. (2006) found increased and more diffuse functional connectivity between the caudate nucleus and cortical areas such as prefrontal cortex, premotor areas, and parietal cortex in adults with ASD during a task of visuomotor coordination. Di Martino et al. (2011) reported increased cortico-striatal resting state connectivity in children with ASD relative to TD children and TD adults, and Delmonte et al. (2013) showed increased resting state connectivity between striatum (specifically caudate and nucleus accumbens) and prefrontal cortex in adults during rest. Di Martino et al. (2011) also reported aberrant as well as increased cortico-striatal connectivity in ASD children relative to TD children and TD adults. These differences were widespread but included limbic regions such as the insula and face processing regions such as the superior temporal cortex. Another prior resting state study reported hypo-connectivity between the ventral striatum and the temporal lobe in children with ASD (Abrams et al., 2013). Taken together, these findings suggest that striatal connectivity is atypical in ASD, and may be predominantly characterized by hyperconnectivity with cortical areas. To date, no study has examined the development of striatal functional connectivity in ASD from childhood to adulthood.

The current study utilized resting state fMRI, expanding on previous work (Di Martino et al., 2011), to explore the development of the functional connectivity of the striatum in ASD and TD. We predicted that TD individuals would show decreasing cortico-striatal connectivity over development, consistent with previous literature (Supekar et al., 2009; Dosenbach et al., 2010). Given prior evidence of relative hyper-connectivity with striatum in both children and adults (Di Martino et al., 2011; Delmonte et al., 2013), we predicted that ASD individuals would overall show increased connectivity relative to typical individuals when controlling for age. We also predicted that ASD individuals would show both deviant and arrested development in connectivity with areas that change typically, especially with regions of the striatum known to support cognitive (dorsal caudate) and affective (ventral striatum) circuits, which may contribute to known behavioral impairments of the disorder. We did not have any directional hypotheses for age related differences in striatal connectivity between ASD and TD given the lack of prior developmental research in this area.

## Materials and methods

### Participants

Forty-two ASD and 48 TD participants between the ages of 8 and 36 were tested. There were no differences in age (*p* = 0.88) and IQ (above 80; *p* = 0.81) between groups. Participants were recruited through the University of Pittsburgh Autism Center of Excellence (ACE) subject core (HD#055748). Participants were diagnosed with ASD using the Autism Diagnostic Interview (ADI; Lord et al., 1994) and Autism Diagnostic Observation Schedule-G (ADOS; Lord et al., 2000). Participants and/or their legal guardians provided consent and assent prior to being enrolled in the study, which was approved by the Internal Review Board at the University of Pittsburgh. The ASD group met cut-offs for autism on the ADI (except one individual in section D) and cut-offs for either autism or spectrum disorder on the ADOS, and an expert clinician confirmed diagnosis. Individuals were excluded from the ASD group if they reported concussions, vision problems, drug abuse, epilepsy, meningitis, and/or encephalitis. There were no effects of age on ADI scores or the ADOS social or final scores (*p*'s > 0.05, Bonferroni corrected for multiple comparisons) though there was a significant effect of age on the ADOS communication score (*t* = 3.422, *p* = 0.002), increasing in severity with age. TD participants were recruited through the Autism Center for Excellence (Pittsburgh, PA) subject core. Exclusion criteria included learning disabilities and psychiatric disorders (individual and first-degree relative). All participants were screened for MR safety (absence of any metal and claustrophobia). See Table 1 for full description of participant demographics.

**Table 1:** **Demographic information**.

	**ASD (***n*** = **41**)**			**TD (***n*** = **48**)**			
Males, *n* (%)	35 (85)			41 (85)			
Right handed, *n* (%)	36 (88)			46 (96)			
							***t*-test**
	**Mean (SD**)	**Minimum**	**Maximum**	**Mean (SD**)	**Minimum**	**Maximum**	***p*-value**
Age (years)	17.28 (6.10)	9.33	33.90	17.08 (6.41)	8.39	36.38	0.88
Full scale IQ	111 (12)	86	131	111 (10)	86	130	0.81
Verbal IQ	108 (11)	83	132	108 (11)	88	132	0.91
Performance IQ	112 (12)	86	128	110 (10)	86	132	0.65
**ADOS**							
Communication	4 (1)	2	8	–			
Social	8 (2)	5	12	–			
Total	12 (3)	7	19	–			
**ADI**							
Social	21 (5)	8	28	–			
Communication	16 (4)	9	25	–			
RRB	6 (2)	2	12	–			
Abnormal	3 (1)	0	5	–			

ADOS, Autism Diagnosis Observation Schedule; ADI, Autism Diagnostic Interview; SD, Standard Deviation; ASD, Autism Spectrum Disorder; TD, Typical Development. IQ, Intelligence Quotient; RRB, Restricted Repetitive Behaviors.

### Procedure

All scans were conducted at the Neuroscience Imaging Center at the McGowan Institute for Regenerative Medicine at the University of Pittsburgh on a Siemens Allegra 3T MRI scanner. Participants first completed six runs of face and car memorization and recognition tasks. Next, we acquired Magnetization-prepared rapid gradient echo (MPRAGE) and DTI sequences prior to the final resting state scan. The participants watched a movie of their choice during structural scans in order to reduce the potential for head movement. During the resting state scan, participants were instructed to lie in the scanner with their eyes closed but to remain awake. Functional images were obtained using a gradient echo, echo-planar imaging (EPI) sequence sensitive to blood-oxygen-level-dependent (BOLD) contrast (*TR* = 1500 ms, *TE* = 25 ms, flip angle = 70°, 29 4 mm slices axial slices, voxel size = 3.1 × 3.1 × 4, 200 volumes per run, *FOV* = 200 mm), interleaved slices MPRAGE sequences (*TR* = 2100 ms, *TE* = 3.93 ms, flip angle = 7°, 176 1 mm axial slices, voxel size = 1.1 × 1.1 × 1.1) were used to obtain structural images, prior to functional imaging.

### fMRI preprocessing

Each participant's resting scan data were motion corrected using AFNI (Cox, 1996), using the first volume as a reference. To correct for signal corrupted by physiological noise, Physiologic EStimation by Temporal Independent Component Analysis (PESTICA v2.0) (Beall and Lowe, 2007) was used to create respiration and cardiac estimators, and apply impulse response function retrospective correction of physiological motion effects (IRF-RETROICOR) (Beall, 2010). These estimates were then filtered temporally based on the empirically derived default windows of 48–85 bpm for cardiac and 10–24 bpm for respiration and adjusted for dithering. Resulting images were then slice time corrected, aligned to the MPRAGE using FLIRT in FSL (Smith et al., 2004) and, scaled to the mean of each voxel. We used Freesurfer's automated segmentation program (Fischl et al., 2002) to segment gray matter, white matter, ventricles and non-brain tissue (NBT) in each participant's MPRAGE scan. These anatomical parcellations were used to extract signal from white matter, ventricles and NBT in the resting state fMRI scans. Using measures of head movement obtained from motion correction, we averaged translation and rotation values in the x, y, and z directions to calculate root mean square (RMS) of linear and angular precision. Next, using the ANATICOR program in AFNI (Jo et al., 2010), we reduced noise and artifacts from hardware, the draining vessel effect, and motion in each gray matter voxel by regressing out the following nuisance variables: (1) motion regressors for the standard 6 parameters, (2) local white matter regressors averaged from white matter voxels within a spherical mask (radius = 30 mm) centered at each gray matter voxel of interest, (3) ventricle signal regressors, and (4) NBT regressors. There were no significant differences in head motion across age (*P* = 0.15) or between groups (*P* = 0.25). Data were subsequently bandpass filtered at 0.009 Hz < f < 0.08 Hz and voxels were spatially smoothed using a 5 mm full width at half maximum Gaussian kernel. Structural scans (MPRAGE) were warped to a standard template space using a template brain from the Montreal Neurological Institute (MNI, Montreal, Canada) using FSL's non-linear registration procedure (FLIRT and FNIRT), and resulting warp coefficients were saved. Preprocessed fMRI data were spatially aligned and normalized to each participant's warped MPRAGE scan using FSL's non-linear registration procedure (FNIRT). Further, using the methods proposed by Power et al. (2012), we calculated frame-wise displacement (FD) and RMS variance of the temporal derivative of the time-series (DVARS). FD and DVARS values were used to identify volumes in the fMRI time series to remove from data analysis. There were no differences in FD between groups or across age (Figure S1) (*p*'s > 0.05). Using the same threshold as Power et al. (2012) we removed volumes where FD exceeded 0.5 mm and DVARS exceeded 0.5% signal change. There were no significant differences between groups (*t* = 0.656, *p* = 0.513) or across age (*t* = −1.449, *p* = 0.151) for the number of volumes removed (TD: mean 26.08 ± 10.02, ASD: mean 30.66 ± 9.38).

### Striatal volume analysis

Given prior research suggesting that the structural development of the striatum differs in ASD relative to TD, we obtained volumetric measurements from the caudate, putamen, and nucleus accumbens, and the whole brain using Freesurfer's automated segmentation tool (Fischl et al., 2002). We entered these values into linear regression models with age and age^2^ as continuous variables, and diagnosis as a categorical variable. We chose to model both linear and quadratic functions of age given prior evidence that striatal structures show both linear and quadratic changes over development (Langen et al., 2009). We also examined whether striatal volume differed as a function of age or diagnosis therefore potentially introducing a confound. We found no age or diagnosis, group main effects or interactions on striatal volume for any of the six striatal structures even at an uncorrected threshold of *p* < 0.05.

### fMRI data analysis

We used functionally distinct seed regions of interest (ROI) in striatum, bilaterally, including the dorsal caudate (DC), inferior and superior ventral striatum (VSi, VSs), dorsal-caudal and dorsal-rostral putamen (dcP, drP), and ventral rostral putamen (vrP) as previously defined in the literature (Di Martino et al., 2008) (Table 2). Prior research using these seed regions has demonstrated increased connectivity in ASD children relative to TD children and adults, suggesting that developmental differences might exist (Di Martino et al., 2011). Striatal ROI's were manually inspected against each participant's warped MPRAGE to ensure that they all fell within the boundaries of the striatal regions that they represented. In each participant's resting scan images, using single value decomposition in AFNI (1dSVD), we extracted the first principal component vector in the time series for each of the 12 ROIs using AFNI (Cox, 1996). In order to ensure that the first component time series was the most representative of all of the voxels in the seed, we correlated this time series with the average time series of each seed. Across participants and across all seed ROI's, the correlation values were above *r* = 0.99. We then correlated each first component vector with time series in every voxel in the brain. Resulting whole brain correlation maps were Z-transformed (Fisher r to z transformation) and entered into group-level regression models with diagnosis (ASD, TD) as a categorical factor and age as a continuous factor, controlling for sex. We also ran regression analyses to examine any non-linear relationships with age, including a quadratic model of age (age^2^), given prior research suggesting that striatal volume in ASD showed both quadratic and linear change with age (Langen et al., 2009). We generated maps of regions that exhibited positive and negative connectivity for each seed as a function of age within group (ASD, TD) as well as age by group interactions. We used a group inclusive mask, masking for regions that overlapped across all participants (Figure S2). To correct for multiple comparisons (family-wise error correction), we ran a Monte-Carlo simulation to determine cluster size at a voxel threshold of *p* = 0.005, and a cluster threshold of *p* = 0.004. We chose the value for the cluster threshold based on a Bonferroni correction for multiple seed regions (*p* = 0.05/12 seeds = 0.004).

**Table 2:** **Coordinates for striatal seed regions in left and right hemisphere in MNI space**.

**Striatal seed**	***x***	***y***	***z***
DC	±13	15	9
dcP	±28	1	3
drP	±25	8	6
vrP	±20	12	−3
VSi	±9	9	−8
VSs	±10	15	0

DC, Dorsal Caudate; dcP, dorsal caudal Putamen; drP, dorsal rostral Putamen; vrP, ventral rostral Putamen; VSi, Ventral Striatum inferior; VSs, Ventral Striatum superior.

Furthermore, as a secondary analysis, we excluded all participants over the age of 25, as we had fewer participants who exceeded this age, and reran the whole brain group analysis. We thus report resulting significant clusters that showed main effects of age or age^2^, main effect of diagnosis group (controlling for age), and age by diagnosis group interaction, across all participants in regions that remained significant when we excluded participants over the age of 25 (in order to ensure that our age effects were not driven by the older participants). For the regions that showed an age by diagnosis interaction, average beta values were extracted for each individual and entered into linear regression models using the lm function in R (R Core Team, 2012) to determine the direction of the developmental slope of each group. To assess the effect of autism diagnosis on connectivity, we ran separate regression models on the ASD group with age and ADI scores as independent variables (controlling for sex) in each of our resulting significant clusters. Lastly, given the recent discussions in the literature regarding the optimal methods of motion correction (e.g., Power et al., 2013; Satterthwaite et al., 2013), we reran our analyses without the motion censoring procedure and covaried FD at the group level.

## Results

For the analyses that included the quadratic function of age, we found no significant clusters. Therefore, all results reported below are based on regression models that included only linear relationships with age. We also did not find any significant effects of ADI scores on connectivity (all *p*'s > 0.05). Lastly, we did not find differences in which clusters were significant when covarying FD at the group level. Therefore, we report our findings when employing the scrubbing procedure described previously.

**Figure 2:**
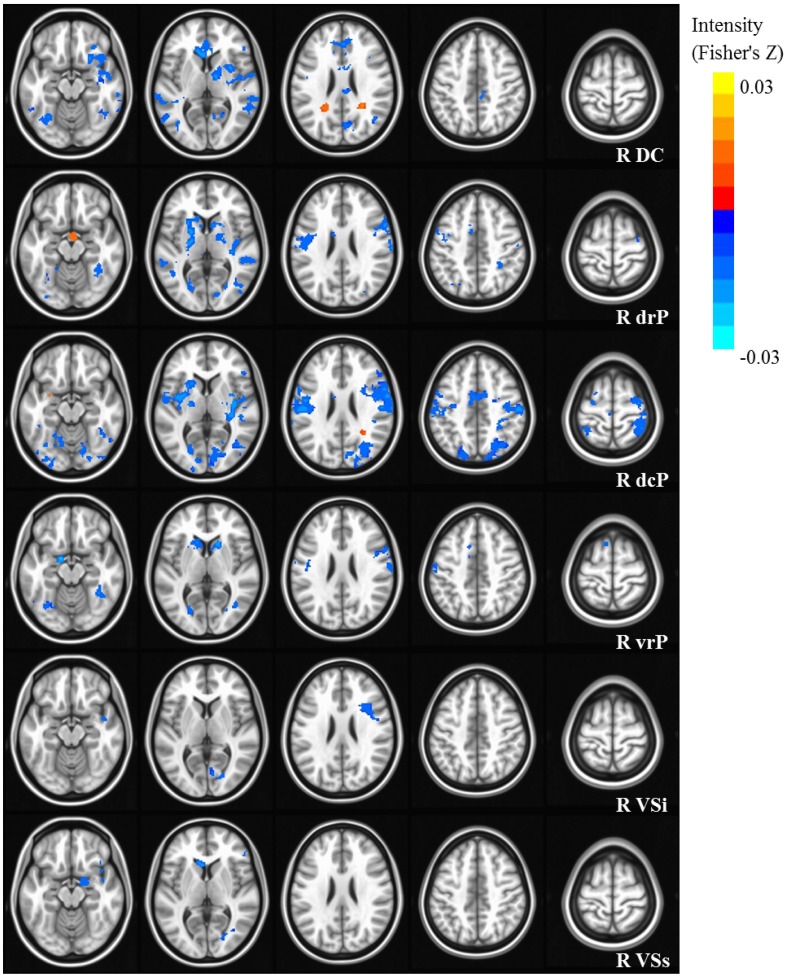
**Statistical maps depicting linear effects of age with right hemisphere seeds**. For all analyses, we used a Monte Carlo simulation for cluster correction (voxel-wise *p* < 0.005, cluster-level *p* < 0.004 or 105 voxels) (AFNI; 3dClustSim). Slices were generated using AFNI software. Blue denotes areas that decreased with age, and orange denotes areas that increase with age. L, Left; R, Right; DC, Dorsal Caudate; dcP, dorsal caudal Putamen; drP, dorsal rostral Putamen; vrP, ventral rostral Putamen; VSi, Ventral Striatum inferior; VSs, Ventral Striatum superior.

**Figure 3:**
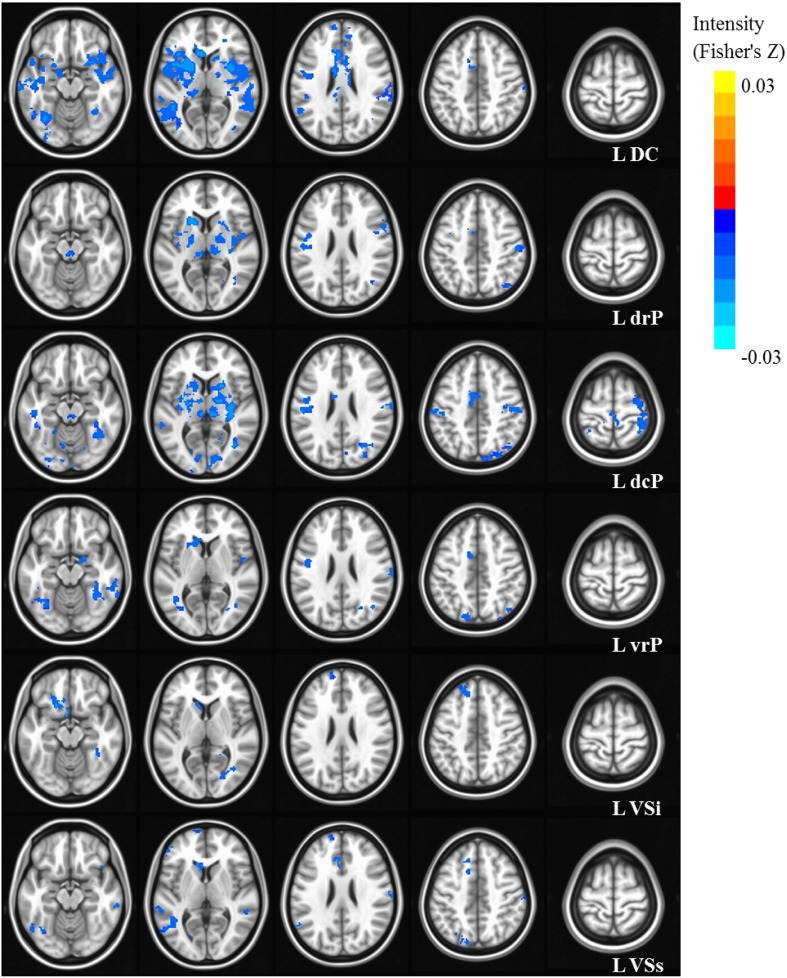
**Statistical maps depicting linear effects of age with left hemisphere seeds**. For all analyses, we used a Monte Carlo simulation for cluster correction (voxel-wise *p* < 0.005, cluster-level *p* < 0.004 or 105 voxels) (AFNI; 3dClustSim). Slices were generated using AFNI software. Blue denotes areas that decreased with age, and orange denotes areas that increase with age. L, Left; R, Right; DC, Dorsal Caudate; dcP, dorsal caudal Putamen; drP, dorsal rostral Putamen; vrP, ventral rostral Putamen; VSi, Ventral Striatum inferior; VSs, Ventral Striatum superior.

**Figure 4:**
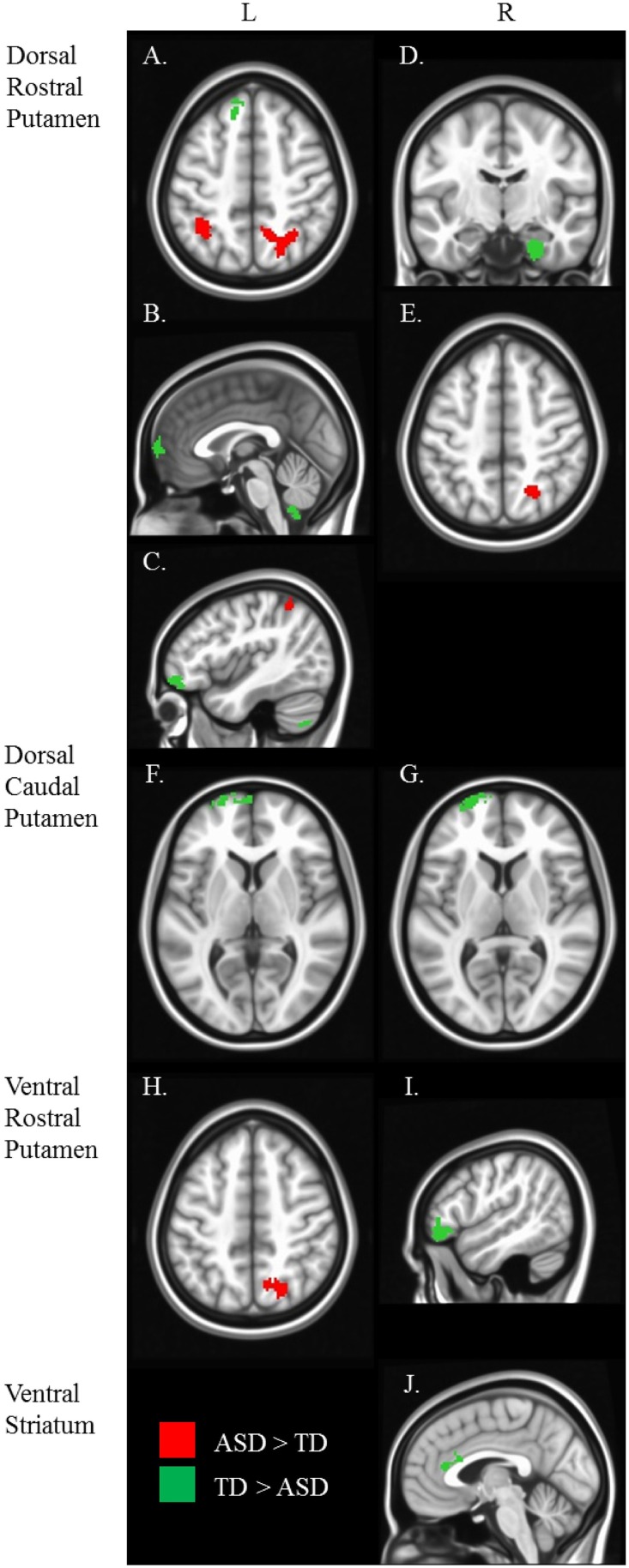
**Between diagnosis group statistical map grouped by striatal region.** For all analyses, we used Monte Carlo simulation for cluster correction (voxel-wise *p* < 0.005, cluster-level *p* < 0.004 or 105 voxels) (AFNI; 3dClustSim). Slices were generated using Analysis of Functional NeuroImages (AFNI) software. Regions showing increased connectivity in ASD relative TD are depicted in red and regions showing increased connectivity in TD relative to ASD are depicted in green. See Table 3 for cluster coordinates and connections to specific seed regions. **(A–E)** Regions connected with the dorsal rostral putamen (drP). **(F–G)** Regions connected with the dorsal caudal putamen (dcP). **(H–I)** Regions connected with the ventral rostral putamen (vrP). **(J)** Regions connected with the inferior ventral striatum (VSi). L, Left Hemisphere. R, Right Hemisphere.

**Figure 5:**
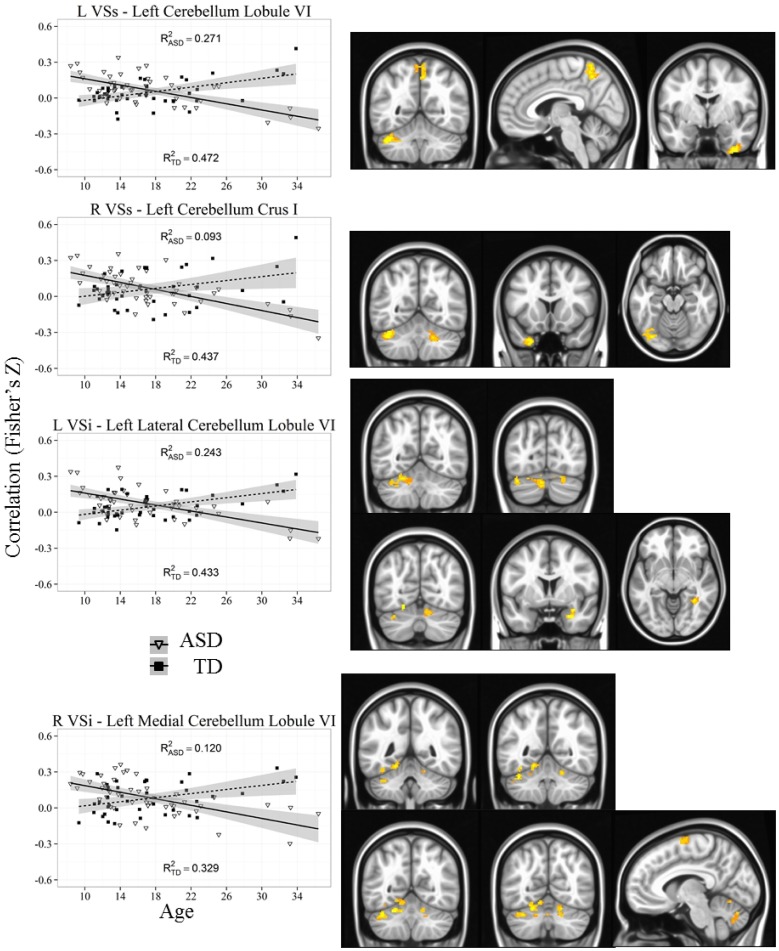
**Representative graph depicting most significant region that showed an age by diagnosis group interactions with ventral striatum (VSs and VSi).** For all analyses, we used a Monte Carlo simulation for cluster correction (voxel-wise *p* < 0.005, cluster-level *p* < 0.004 or 105 voxels) (AFNI; 3dClustSim). Z-transformed correlation coefficients are displayed on the y-axis and age in years on the x-axis of each graph. Title of each graph describes the seed region and the relevant connecting cluster. Triangles and solid lines are TD participants, squares and dashed lines are ASD participants. L, Left; R, Right; VSi, Ventral Striatum inferior; VSs, Ventral Striatum superior; TD, Typical Development; ASD, Autism Spectrum Disorder. See Table 4 for cluster coordinates. See Figure S5 for all graphs.

**Figure 6:**
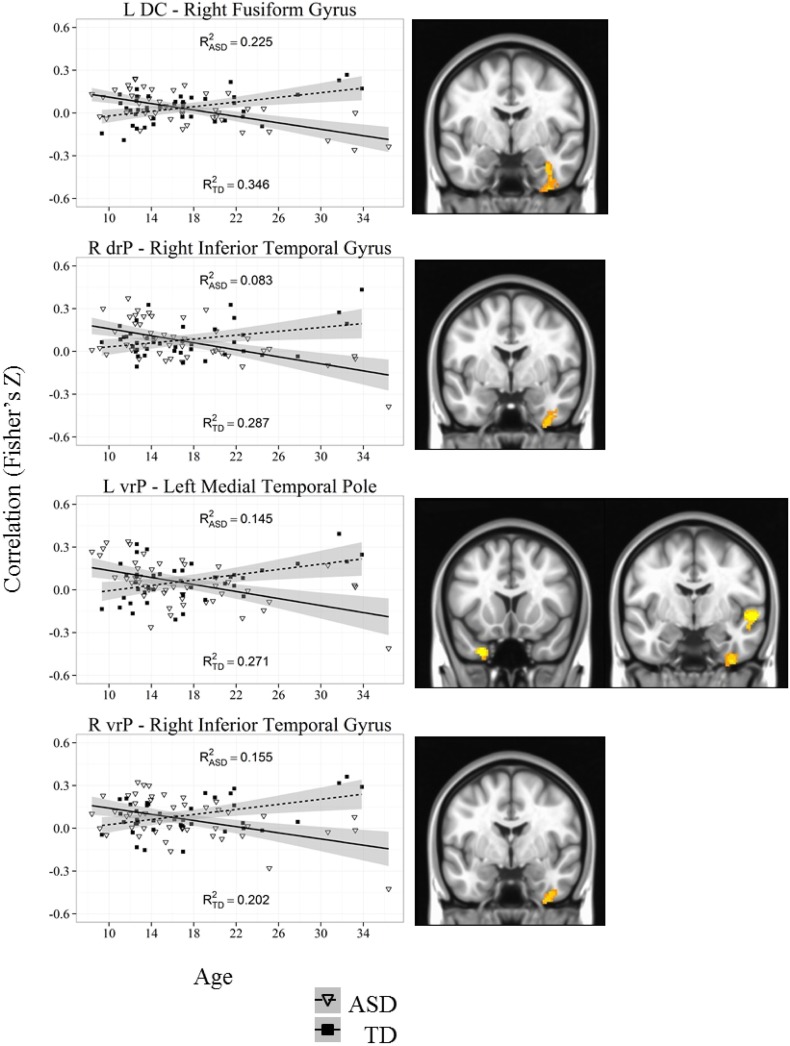
**Representative graph depicting most significant region that showed an age by diagnosis group interactions with caudate and putamen (DC, drP, vrP).** For all analyses, we used a Monte Carlo simulation for cluster correction (voxel-wise *p* < 0.005, cluster-level *p* < 0.004 or 105 voxels) (AFNI; 3dClustSim). Z-transformed correlation coefficients are displayed on the y-axis and age in years on the x-axis of each graph. Title of each graph describes the seed region and the relevant connecting cluster. Partial *R*^2^ values of the regression lines for each group are depicted on each graph (*R*^2^_ASD_ and *R*^2^_TD_). Triangles and solid lines are TD participants, squares and dashed lines are ASD participants. L, Left; R, Right; DC, Dorsal Caudate; drP, dorsal rostral Putamen; vrP, ventral rostral Putamen; TD, Typical Development; ASD, Autism Spectrum Disorder. See Table 4 for cluster coordinates. See Figure S5 for all graphs.

Our results were consistent with previous work on striatal connectivity and development. Collapsed across groups and age, we found patterns of positive correlations between the striatal seeds and a distributed set of cortical areas. Overall, connectivity patterns were similar to previously published research in TD adults (Di Martino et al., 2008) (Figures S3, S4). Independent of diagnostic group, we found decreases with age in connectivity between striatal seeds and a wide set of striatal and cortical regions including prefrontal, temporal and parietal cortices, and cerebellum (Figures 2, 3). Below, for each striatal seed, we first report clusters that showed a main effect of diagnosis group when controlling for age, to establish regions that show differences in ASD relative to TD overall (Figure 4, Table 3). We then report clusters that showed significant group by age interactions (Figures 5, 6, and Figure S5, Table 4).

**Table 3:** **Regions showing a significant main effect of diagnosis group, controlling for age**.

**Region**		**Volume**	**Center mass**			**Mean**	**SEM**	**Max intensity**
**Seed**	**Cluster**		***X***	***Y***	***Z***			
R VSi	Left anterior cingulate cortex	1056	3	−26.4	20.9	0.1752	0.0011	−0.2132
R dcP	Left middle frontal gyrus	1376	17.5	−65.8	6.9	0.1611	0.0017	−0.2035
L dcP	Left superior medial gyrus	1816	10.7	−66.1	3	0.1682	0.002	−0.2207
R drP	Right parahippocampal gyrus	1296	−26	11.4	−30.4	0.1742	0.0018	−0.2283
	Right superior occipital gyrus	896	−22.3	62.4	46.7	0.1906	0.0016	0.2202
L drP	Right superior parietal lobule	3032	−18	60.6	49.2	0.175	8.70E-04	0.2178
	Left inferior parietal lobule	2160	34.9	50.2	51.6	0.1916	0.0012	0.2457
	Left superior medial gyrus	1016	1.8	−64.8	5.5	0.1715	0.0024	−0.224
	Left superior frontal gyrus	944	11.5	−38.4	47.5	0.1693	0.0017	−0.2155
	Left inferior frontal gyrus	880	41.3	−49.3	−12.4	0.1878	0.0018	−0.2279
R vrP	Right inferior frontal gyrus	1200	−51.4	−37.4	−8.4	0.207	0.0013	−0.2524
L vrP	Right superior parietal lobule	1640	−16.9	66.4	50.1	0.182	0.0014	0.2337

L, Left; R, Right; DC, Dorsal Caudate; dcP, dorsal caudal Putamen; drP, dorsal rostral Putamen; vrP, ventral rostral Putamen; VSi, Ventral Striatum inferior; VSs, Ventral Striatum superior.

Negative max intensity values indicate that TD showed increased connectivity relative to ASD. Positive max intensity values indicate that ASD showed increased connectivity relative to TD.

**Table 4:** **Regions showing significant Age × Diagnosis Group Interactions**.

**Region**		**Volume**	**No. Voxels**	**Center mass**			**Maximum Intensity**	**ASD R^2**	**TD R^2**
**Seed**	**Cluster**			***X***	***Y***	***Z***			
L DC	Right fusiform gyrus	1752	219	−34.5	3.6	−38.9	0.0254	0.2247^**^	0.3456^***^
R drP	Right inferior temporal gyrus	1296	162	−37.4	1.7	−42.2	0.0265	0.08304^*^	0.2872^***^
L vrP	Left medial temporal pole	1192	149	28.2	−13.7	−36.8	0.0294	0.1446^**^	0.2713^***^
	Right fusiform gyrus	1128	141	−35.8	3.2	−45.1	0.0261	0.1556^**^	0.2585^***^
	Right superior temporal gyrus	912	114	−55.9	7	−3.6	0.0328	0.0372	0.2445^***^
R vrP	Right inferior temporal gyrus	1024	128	−38.1	3.1	−44.1	0.0244	0.1551^**^	0.2022^**^
L VSi	Left cerebellum, lobule VI	4464	558	32.2	58.7	−27.4	0.0305	0.2433^***^	0.4331^***^
	Left cerebellum, lobule VI	1384	173	8.2	76.2	−26.8	0.0272	0.1905^**^	0.2116^***^
	Right cerebellum, lobule VI	1040	130	−15.9	70.7	−22.3	0.0241	0.1866^**^	0.1588^**^
	Right amygdala	912	114	−26.5	−1.6	−23	0.0299	0.2456^***^	0.1401^**^
	Right fusiform gyrus	840	105	−35.6	47.4	−7.3	0.0268	−0.0040	0.4629^***^
R VSi	Left cerebellum, lobule VI	3344	418	17.6	55.8	−16.4	0.031	0.1205^*^	0.3287^***^
	Right cerebellum, lobule VI	2128	266	−15.3	65.9	−22.3	0.0272	0.1976^**^	0.1996^***^
	Left cerebellum, lobule VI	1904	238	36.6	53.6	−28.1	0.0301	0.1436^**^	0.371^***^
	Left cerebellum, lobule VIIIa	1456	182	3.4	70.8	−33.6	0.0266	0.3367^***^	0.1173^*^
	Left supplementary motor area	1104	138	5.1	7.1	69.1	0.0261	0.1789^**^	0.2765^***^
L VSs	Left cerebellum, lobule VI	5192	649	34.7	54.9	−30.7	0.0349	0.2705^**^	0.4721^***^
	Right precuneus	2104	263	−4.5	52.9	58.4	0.0292	0.1429^**^	0.2224^**^
	Right inferior temporal gyrus	2088	261	−37.7	3.4	−44.5	0.0333	0.2006^**^	0.3412^***^
R VSs	Left cerebellum, crus I	2752	344	40.6	55.8	−29.5	0.0304	0.09309^*^	0.4372^***^
	Left medial temporal pole	1528	191	29	−14.1	−37.1	0.0296	0.1208^*^	0.3211^***^
	Left fusiform gyrus	1248	156	35.8	75.6	−17.3	0.0285	0.08311^*^	0.2785^***^
	Right cerebellum, lobule VI	856	107	−19.9	59.4	−29.9	0.0253	0.3396^***^	0.1681^**^

* p < 0.05;

** p < 0.01;

*** p < 0.001. L, Left; R, Right; DC, Dorsal Caudate; dcP, dorsal caudal Putamen; drP, dorsal rostral Putamen; vrP, ventral rostral Putamen; VSi, Ventral Striatum inferior; VSs, Ventral Striatum superior.

### Dorsal caudate (DC)

The DC has extensive connections with dorsal and lateral aspects of cortex involved in inhibitory control, working memory, and task switching.

Group differences: We found no group differences between the DC when controlling for age.

Whole brain group × age interaction: We found significant age by diagnosis interactions in connectivity between the left DC and right fusiform gyrus. TD individuals exhibited a significant decrease in connectivity with age, and ASD individuals showed increased connectivity with age.

### Dorsal caudal and rostral putamen (dcP, drP)

The dorsal putamen is involved in primary motor control including motor selection and execution.

#### dcP

Group differences: We found a main effect of diagnosis group in connectivity between the left dcP and the left superior medial gyrus, and between the right dcP and the left middle frontal gyrus. In both clusters, TD individuals exhibited increased connectivity relative to ASD.

#### drP

Group differences: With the left drP, the ASD group showed increased connectivity with the right superior and inferior parietal lobule, and decreased connectivity with the left superior medial, superior frontal, and inferior frontal gyri. With the right drP, the ASD group demonstrated decreased connectivity with the right parahippocampal gyrus, and increased connectivity with the superior occipital gyrus.

Whole brain group × age interaction: We found no whole brain age group by diagnosis interactions with either the dcP or the drP.

### Ventral rostral putamen (vrP)

The ventral rostral putamen is implicated in cognitive control and executive function, with connections to the anterior cingulate, and regions of the insula.

Group differences: Collapsed across age, the ASD group showed increased connectivity between the left vrP and right superior parietal lobule, and decreased connectivity between the right vrP and the right inferior frontal gyrus, relative to TD.

Whole brain group × age interaction: There were significant age by diagnosis interactions in connectivity between the left vrP and the left medial temporal pole, the right fusiform gyrus, and the right superior temporal gyrus. We also found an interaction between the right vrP and the right inferior temporal gyrus. The ASD group showed increased connectivity with age, and the TD group showed significant decreases with age in all clusters except the superior temporal gyrus, which showed no change over development in the ASD group and a significant decrease in connectivity in the TD group.

### Inferior and superior ventral striatum (VSi and VSs)

The ventral striatum, which has connections to medial aspects of the prefrontal cortex, and other areas of the limbic system, is strongly implicated in reward-related processing.

#### VSi

Group differences: We found a significant group difference between the Right VSi and the right anterior cingulate cortex.

Whole brain group × age interaction: Age by diagnosis group interactions were evident in connectivity from both VSi seeds to the bilateral cerebellum (Lobule VI and Lobule VIIa, Crus I). In addition, we found an interaction between the left VSi and the right amygdala and right fusiform gyrus, and between the right VSi and the left supplementary motor area. The ASD participants showed significant increases with age whereas TD showed decreases with age except with the right fusiform gyrus, where the ASD group showed no change with development.

#### VSs

Whole brain group × age interaction: There were significant age by diagnosis group interactions between both VSs seeds and the cerebellum (Lobules VI and VIIa, Crus I), precuneus, and right inferior temporal gyrus. The ASD group showed increased connectivity with age, whereas the TD group showed decreased connectivity with age.

## Discussion

We examined striatal resting state functional connectivity across the ages of 8–36 years in individuals diagnosed with ASD, relative to typically developing individuals. Using previously defined striatal seed ROIs (Di Martino et al., 2008), we identified connections associated with each seed, respectively, and examined changes in connectivity patterns across age and between diagnosis groups. To the best of our knowledge this study is the first to examine striatal functional connectivity in ASD across development, from late childhood to adulthood. Thus, our results provide a novel understanding of the development of functional connectivity with the striatum in ASD and identify connectivity patterns that parallel and deviate from typical development.

Patterns of striatal functional connectivity in both individuals with ASD and TD individuals were consistent with previous studies utilizing the same seed regions in both child and adult populations separately (Di Martino et al., 2008, 2011; Kelly et al., 2009b; Furman et al., 2011). In general, we noted a dorsal to ventral and medial to lateral gradient, where more dorsal seeds in striatum were significantly connected to dorsal and lateral aspects of cortex, and ventral areas of striatum connected to more medial and ventral cortical regions. This is consistent with purported cognitive/affective divisions previously identified in cortico-striatal circuits using the same seed regions (Di Martino et al., 2008). Collapsed across diagnostic groups, we found decreases in connectivity between all striatal seeds and various cortical areas across age, consistent with studies of typical development, which may reflect necessary decreases in striatal influence over cortical function, supporting the emergence of long-range cortico-cortico connectivity in adulthood (Fair et al., 2007, 2009; Kelly et al., 2009a; Supekar et al., 2009; Dosenbach et al., 2010). As developmental changes that occur in typically developing individuals are often interpreted as necessary for maturation into adulthood, regions that show similar age-related change with TD suggest intact development in ASD. Thus, deviations from typical maturation may indicate development of compensatory connections or impairments that persist into adulthood.

Stable disorder effects (i.e., connections that are atypical in ASD independent of age) were noted with only the putamen and the inferior ventral striatum seeds, and suggest that posterior connections (superior and inferior parietal lobule) are increased, whereas anterior connections (anterior cingulate and superior, middle, and inferior frontal gyri) are decreased in ASD. Previous resting state functional connectivity studies using the striatum did not find differences in the posterior parietal cortex, and differences with the prefrontal cortex have been in the opposite direction with ASD individuals (both children and adults) showing increased striatal-prefrontal connectivity relative to typicals (Turner et al., 2006; Di Martino et al., 2011; Delmonte et al., 2013) with some studies reporting no differences (Kennedy and Courchesne, 2008; Tyszka et al., 2013). Discrepant findings previously might have been specific to the age groups tested, whereas the present study controlled for age-related changes when examining group differences. We suggest that there are differences in network connectivity in ASD that is characterized by both hypo- and hyper- connectivity in a region specific manner. Although the behavioral implications of these findings are unclear, these novel results highlight atypical connectivity patterns that are unchanged with development (see Figure 1B). One caveat to the interpretation of these group level findings in relation to prior results must be highlighted. We did not include the average global time series as a nuisance regressor (GSR), as prior studies did due to our use of the PESTICA program to estimate and remove the effects of physiological noise, as well as the recent evidence in the literature regarding the potential spurious correlations that may arise when using GSR with seed based resting state analyses in both typical (Saad et al., 2012), and ASD samples (Gotts et al., 2013). A recent resting state study in children with ASD suggested that GSR does not significantly alter resting state results (Di Martino et al., 2013), although those findings were specific to a different measure of connectivity [network centrality—which may be less affected by GSR (Yan et al., 2013)] than the seed based correlations used in the current study. Although differences in preprocessing is a limitation in our ability to compare our results to previous findings, nonetheless, we estimated and removed physiological noise without the potential confounds of GSR. As it is vital that results be comparable in the literature, and future developmental work in ASD should consider both GSR and non-GSR approaches in analysis and interpretation.

The predominant goal of the current study was to identify regions that showed age-related differences from late childhood to adulthood in ASD relative to TD. Within the majority of clusters that showed age by diagnosis group interactions, the TD individuals showed significant developmental decreases, whereas the ASD group showed increases with age, suggesting deviating developmental trajectories into adulthood. Similar to the direction of results in the current study, prior research has suggested that maturation of white matter connectivity is aberrant in ASD relative to TD in a similar fashion in roughly the same age range (10–40), with ASD participants showing increased white matter integrity in subcortical to cortical projection tracts across age, whereas TD participants showed decreased white matter connectivity (Kleinhans et al., 2012). It is possible that age-related differences in structural connectivity in subcortical-cortical tracts underlie the functional differences noted in the present study.

Notably, age by diagnosis interactions revealed that the connectivity between striatum and superior aspects of the cerebellum, specifically with regions VI and VIIa (including Crus I) were decreased in TD participants but increased in ASD. These differences in the development of cerebellar connectivity are not surprising given the convergence of evidence targeting the cerebellum as a locus of abnormality in ASD (Courchesne et al., 1988; Nowinski et al., 2005; for review see Fatemi et al., 2012). The cerebellum has extensive connections with cortical and subcortical brain regions, including bidirectional connections with striatum (Habas et al., 2009; Krienen and Buckner, 2009; Strick et al., 2009; Bostan and Strick, 2010). Studies have previously shown that individuals with lesions in cerebellar areas including lobules VI and VIIa demonstrate cognitive impairments such as motor control and planning, attention, sensory integration, language, and affective processes (Habas et al., 2009; Krienen and Buckner, 2009; Stoodley et al., 2010), all of which are known to be affected in ASD. Furthermore, structural MRI research has found reduced overall cerebellar as well as reduced regional gray matter volume in children, adolescents and adults with ASD (Hashimoto et al., 1995; Bauman and Kemper, 2005; Stanfield et al., 2008; Riva et al., 2013). Functional MRI research has demonstrated atypical cerebellar activation during motor control (Muller et al., 2001; Allen et al., 2004; Mostofsky et al., 2009), and attention (Allen and Courchesne, 2003) in children, adolescent and adults with ASD separately. Finally, functional connectivity findings suggest reduced connectivity between cerebellum and motor execution areas (e.g., sensorimotor and supplementary motor cortices) in children with ASD (Mostofsky et al., 2009). These findings highlight a potential developmental deficit where initial hypoconnectivity relative to TD may be later compensated with relative hyperconnectivity in adulthood, specifically in more ventral aspects of striatum that are involved in reward processing (VSi and VSs). As we did not find any significant correlations with our cerebellar clusters and ADI scores, further work will have to explore the behavioral implications of the aberrant development of cerebellar connectivity in ASD. It is important to note one limitation with our cerebellar results; due to differences in head size between participants, we were unable to acquire complete coverage of cerebellum across all participants. Therefore, our results were limited to the anterior and superior portions of the cerebellum. It is possible that there are connectivity differences with inferior aspects of the cerebellum that we were unable to detect.

The inferior and superior temporal gyri (ITG and STG) and the fusiform gyrus (FG) also showed age by diagnosis group interactions, mainly demonstrating increased connectivity with age in ASD, and decreased in TD. Two clusters suggested developmental arrests or delays in ASD (showing no change with age), and significant decreases with TD; connectivity between the left vrP and the superior temporal gyrus, and the left VSi and the right fusiform gyrus. Structural abnormalities of the temporal gyri gray matter have also been reported in children, adolescents, and adults with ASD, which may contribute to differences in the development of connectivity with temporal regions (Jou et al., 2010; Toal et al., 2010). ITG and FG connectivity differences were found in connections with both dorsal and ventral aspects of the striatum. The ITG and FG, which are components of the ventral stream visual pathway with direct connections to occipital cortex, are largely implicated with face processing, face recognition, and in discrimination of facial expression, including affective interpretation, which may be affected in ASD (Sergent et al., 1992; Kanwisher et al., 1997; Apps et al., 2012; Prochnow et al., 2013). Exaggerated ITG activation and reduced FG activation during face perception in young adults with ASD has also previously been reported (Schultz et al., 2000; Coutanche et al., 2011). The STG has previously been implicated in social communication abnormalities in ASD (Frith, 2001; Wang et al., 2007; Pelphrey et al., 2009; Hubbard et al., 2012). Therefore, abnormalities in striatal connectivity with the temporal cortex may underlie social and/or social reward deficits.

We also found a significant age by group interaction between the inferior ventral striatum and the amygdala. Several prior studies have reported altered activation in and reduced connectivity with the amygdala in association with social perception deficits in ASD (e.g., Kleinhans et al., 2008; Pelphrey and Carter, 2008; Sato et al., 2012), although these findings were with the ITG and not striatum. Given the role of the ventral striatum in reward-related processing and its extensive connections to the amygdala, it is possible that aberrant functional connectivity between striatum and amygdala underlies deficits in social rewards in ASD, which may increase in a compensatory fashion over development (e.g., Delmonte et al., 2012; Sepeta et al., 2012).

This is the first study to examine age related change in functional connectivity with striatum in ASD compared to typical development. We identified a number of connections, with a range of brain regions, showing atypical development from late childhood to adulthood. Importantly, we found that social processing regions such as ITG, STG, and FG, and cerebellar regions implicated in cognitive and motor functions demonstrated a decrease in connectivity over development in TD, but an increase in ASD. As these are novel findings, replication will be necessary, especially given recent debates in the literature regarding methodological considerations related to head motion (e.g., Power et al., 2012; Satterthwaite et al., 2013; Yan et al., 2013), and the removal of nuisance variables such as physiological noise and/or the global signal e.g., when analyzing resting state data. In addition, it is likely that larger sample sizes, wider age ranges, and longitudinal data are needed to replicate these findings, and perhaps identify patterns that the current study may not have had the power to detect, including correlations with symptoms of ASD, identifying regions that show developmental delays, and non-linear trajectories. Despite these limitations, our findings were robust and highlight the important notion that examining the progression of ASD over development is crucial for identifying the neural bases of ASD and how they relate to behavioral impairments in the disorder.

### Conflict of interest statement

The authors declare that the research was conducted in the absence of any commercial or financial relationships that could be construed as a potential conflict of interest.

## Supplementary material

Figure S1 **Scatter plot of mean FD values across groups.** Mean framewise displacement (FD) values for each participant on Y-axis and age in years is depicted on the X-axis. TD participants are in open circles and ASD participants in filled circles. TD, Typical Development; ASD, Autism Spectrum Disorder. There were no significant differences in FD between groups or across age *p* > 0.05.

Figure S2 **Mask of overlapping voxels across all participants**.

Figure S3 **Statistical maps depicting connectivity with striatal seeds in the left hemisphere across all participants, controlling for age**. For all analyses, we used a Monte Carlo simulation for cluster correction (voxel-wise *p* < 0.005, cluster-level *p* < 0.004 or 105 voxels) (AFNI; 3dClustSim). Slices were generated using AFNI software. L, Left; DC, Dorsal Caudate; dcP, dorsal caudal Putamen; drP, dorsal rostral Putamen; vrP, ventral rostral Putamen; VSi, Ventral Striatum inferior; VSs, Ventral Striatum superior.

Figure S4 **Statistical maps depicting connectivity with striatal seeds in the right hemisphere across all participants, controlling for age**. For all analyses, we used a Monte Carlo simulation for cluster correction (voxel-wise *p* < 0.005, cluster-level *p* < 0.004 or 105 voxels) (AFNI; 3dClustSim). Slices were generated using AFNI software. R, Right; DC, Dorsal Caudate; dcP, dorsal caudal Putamen; drP, dorsal rostral Putamen; vrP, ventral rostral Putamen; VSi, Ventral Striatum inferior; VSs, Ventral Striatum superior.

Figure S5 **All graphs showing age by group interactions**. For all analyses, we used a Monte Carlo simulation for cluster correction (voxel-wise *p* < 0.005, cluster-level *p* < 0.004 or 105 voxels) (AFNI; 3dClustSim). Z-transformed correlation coefficients are displayed on the y-axis and age in years on the x-axis of each graph. Title of each graph describes the seed region and the relevant connecting cluster. Triangles and solid lines are TD participants, squares and dashed lines are ASD participants. L, Left; R, Right; DC, Dorsal Caudate; drP, dorsal rostral Putamen; vrP, ventral rostral Putamen; TD, Typical Development; ASD, Autism Spectrum Disorder. See Table 4 for cluster coordinates.
