# Supplementary figures and images for: Age related changes in striatal resting state functional connectivity in autism
Source: Front Hum Neurosci. 2013 Nov 28;7:814. doi: 10.3389/fnhum.2013.00814 (PMC3842522; doi:10.3389/fnhum.2013.00814)

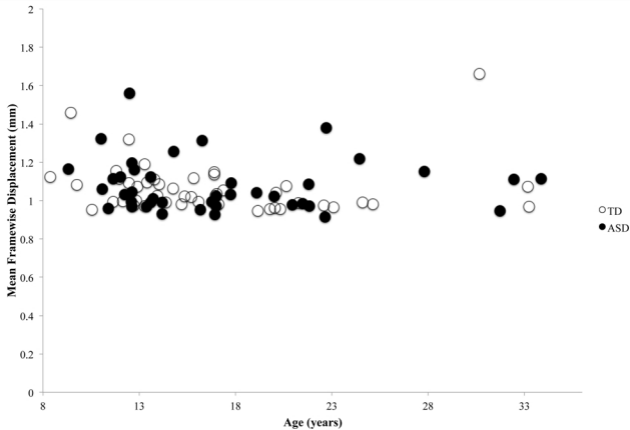

Supplement: Figure S1 — Scatter plot of mean FD values across groups. Mean framewise displacement (FD) values for each participant on Y-axis and age in years is depicted on the X-axis. TD participants are in open circles and ASD participants in filled circles. TD, Typical Development; ASD, Autism Spectrum Disorder. There were no significant differences in FD between groups or across age p > 0.05. [file Presentation1.PDF]

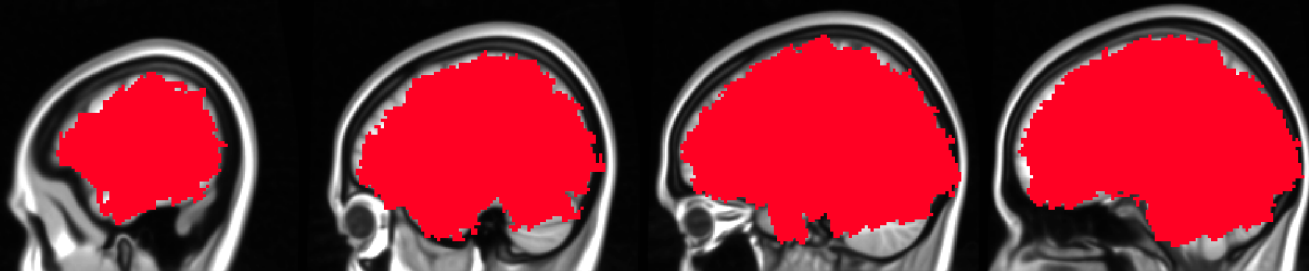

Supplement: Figure S2 — Mask of overlapping voxels across all participants. [file Presentation2.PDF]

Intensity  
(Fisher's Z)

0.75

-0.75

L DC

L drP

L dcP

L vrP

L VS<sub>i</sub>

L VS<sub>s</sub>

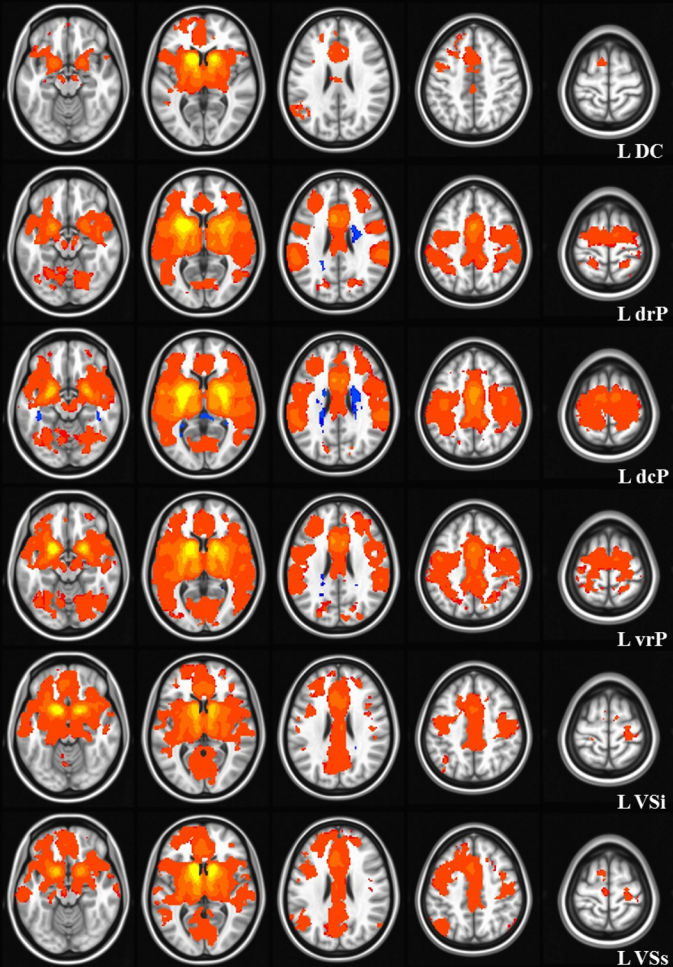

Supplement: Figure S3 — Statistical maps depicting connectivity with striatal seeds in the left hemisphere across all participants, controlling for age. For all analyses, we used a Monte Carlo simulation for cluster correction (voxel-wise p < 0.005, cluster-level p < 0.004 or 105 voxels) (AFNI; 3dClustSim). Slices were generated using AFNI software. L, Left; DC, Dorsal Caudate; dcP, dorsal caudal Putamen; drP, dorsal rostral Putamen; vrP, ventral rostral Putamen; VSi, Ventral Striatum inferior; VSs, Ventral Striatum superior. [file Presentation3.PDF]

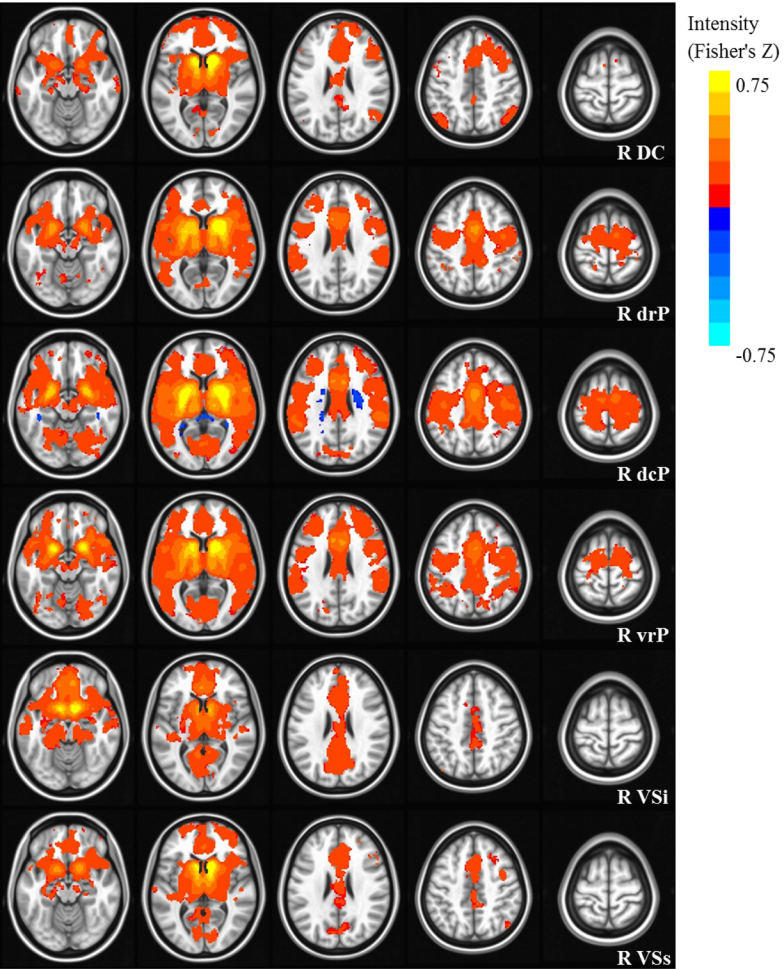

Supplement: Figure S4 — Statistical maps depicting connectivity with striatal seeds in the right hemisphere across all participants, controlling for age. For all analyses, we used a Monte Carlo simulation for cluster correction (voxel-wise p < 0.005, cluster-level p < 0.004 or 105 voxels) (AFNI; 3dClustSim). Slices were generated using AFNI software. R, Right; DC, Dorsal Caudate; dcP, dorsal caudal Putamen; drP, dorsal rostral Putamen; vrP, ventral rostral Putamen; VSi, Ventral Striatum inferior; VSs, Ventral Striatum superior. [file Presentation4.PDF]

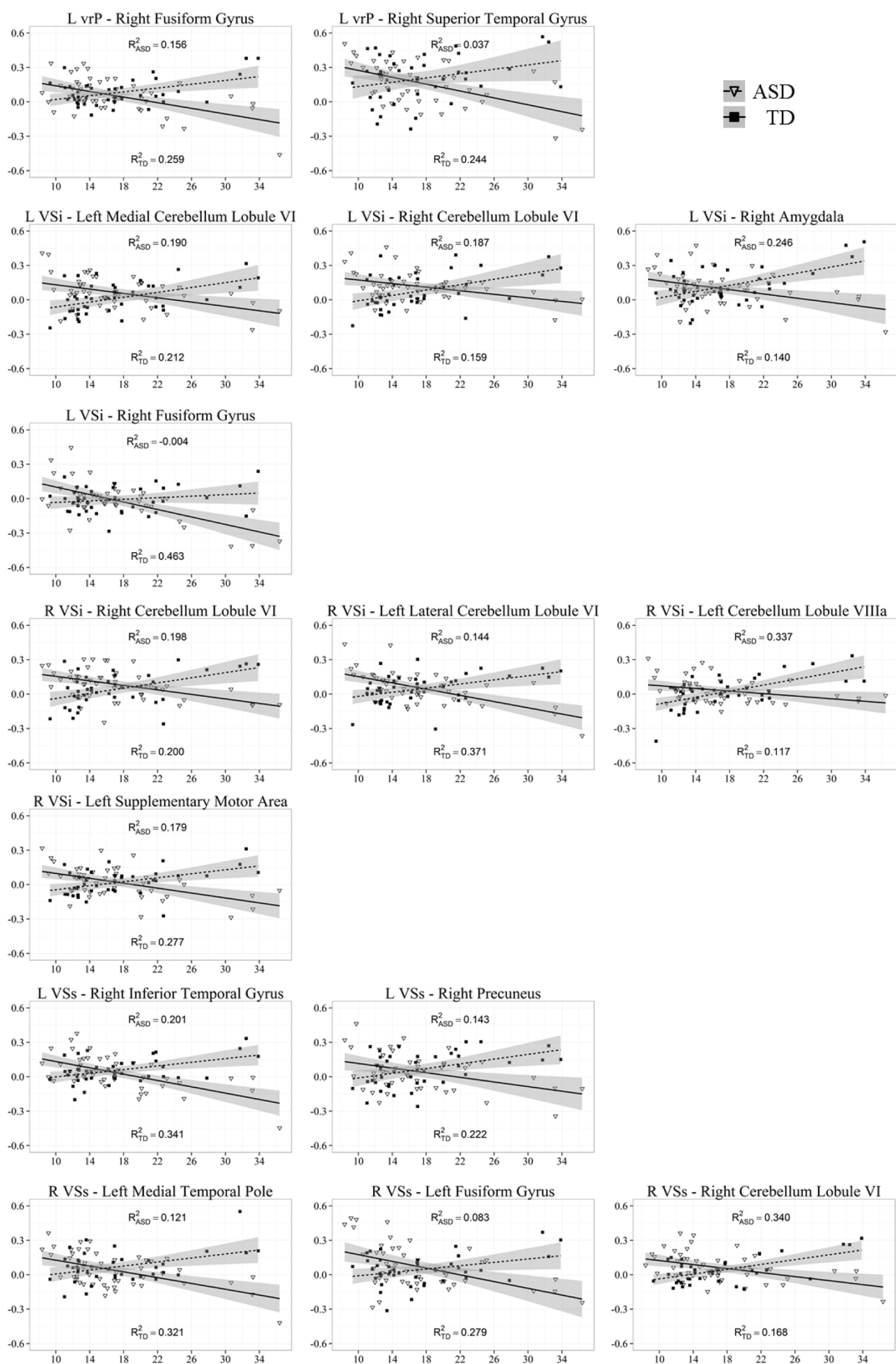

Supplement: Figure S5 — All graphs showing age by group interactions. For all analyses, we used a Monte Carlo simulation for cluster correction (voxel-wise p < 0.005, cluster-level p < 0.004 or 105 voxels) (AFNI; 3dClustSim). Z-transformed correlation coefficients are displayed on the y-axis and age in years on the x-axis of each graph. Title of each graph describes the seed region and the relevant connecting cluster. Triangles and solid lines are TD participants, squares and dashed lines are ASD participants. L, Left; R, Right; DC, Dorsal Caudate; drP, dorsal rostral Putamen; vrP, ventral rostral Putamen; TD, Typical Development; ASD, Autism Spectrum Disorder. See Table 4 for cluster coordinates. [file Presentation5.PDF]
